# Cognitive focus affects spatial decisions under conditions of uncertainty

**DOI:** 10.1007/s10339-020-00952-0

**Published:** 2020-01-23

**Authors:** Thora Tenbrink, Holly A. Taylor, Tad T. Brunyé, Stephanie A. Gagnon, Aaron L. Gardony

**Affiliations:** 1 School of Languages, Literatures and Linguistics, Bangor University, Bangor, UK; 2 Department of Psychology, Tufts University, Medford, MA USA; 3 Department of Psychology, Stanford University, Stanford, CA USA; 4 Cognitive Science, U.S. Army CCDC Soldier Center, Natick, MA USA; 5 Center for Applied Brain and Cognitive Sciences, Tufts University, Medford, MA USA

**Keywords:** Cognitive focus, Spatial cognition, Decision-making, GPS, Uncertainty

## Abstract

Finding one’s way to a destination is a common, everyday task that often relies on spatial information provided by humans and/or automatic devices. However, the information can be inaccurate. How we decide which route to take will depend on our thoughts about the available route information, including who or what provided it, and how these sources may be associated with differential accuracy and fallibility. In three experiments (previously reported in Brunyé et al. (Q J Exper Psychol 68(3):585–607, 2015)), we found that when route directions conflicted with the perceived environment, people trusted the *landmark* information other humans provided, but relied on the *turn direction* information from an automatic device. But what guides these behavioral results? Here we present a systematic linguistic analysis of retrospective reports that sheds some light on how information about the direction source affects cognitive focus. A focus on direction sources in the instruction triggered a cognitive focus on the direction source throughout. Participants who systematically switched strategies focused more on features of the scenario than those who did not. Non-switching strategies were associated with a higher focus on the participants’ own reasoning processes, in particular when relying on turn information. These results highlight how cognitive focus is guided by scenario factors and individual preferences, triggering inferences that influence decisions.

## Introduction

Imagine you are trying to find your way in a city, equipped with route directions. You are supposed to *turn left at the church*, but once you arrive at the church you find that you can only turn right, whereas you could turn left at the supermarket farther ahead. How do you decide which turn to take? What influences your decision, and what do you focus on to guide decision-making? Our previous research (Brunyé et al. 2015) showed that your decision will crucially depend on the nature of the direction source. If the source of your route directions is unknown, you will either go with the turn direction (i.e., decide to turn left even though this means turning at the supermarket rather than the church) or with the landmark (i.e., turn right instead of left at the church), or you might switch between the two approaches. However, if the source is an automatic GPS-based system, you are much more likely to rely on the turn direction—perhaps based on your experience that modern GPS systems typically provide accurate turn guidance but are either not well informed about or not updated with the latest landmarks. And if your source is a human, you will most probably turn at the indicated landmark, even though the resulting turn direction mismatches the instructions. This landmark reliance seems to make sense as humans are known to rely on salient landmarks to guide navigation (Steck and Mallot 2000) but also confuse left and right, or they may have imagined you coming from a different direction.

Strikingly, although the participants’ decisions in Brunyé et al. (2015) seemed to make intuitive sense as just suggested, there is nothing inherent in the different route direction sources that associates landmarks with one source and turn directions with another. In fact, in retrospective reports, people gave inconsistent reasons for making particular choices. Nevertheless, they must have gone through a reasoning process that was sufficiently consistent across participants to lead to the strikingly clear patterns that we found. The information about the direction source must have turned a switch, so to speak, in people’s minds, leading them to consistently consider some aspects over others, such that GPS systems might be associated with reliable turn direction information and human route givers with landmarks. In this paper, we address the nature of this reasoning process and ask what factors might “turn the switch.” To assess the aspects prominent in people’s minds under diverse uncertainty conditions, we present a systematic linguistic analysis of the highly diverse rationales given for decisions—rationales that nevertheless led to homogeneous outcomes.

Participants in Brunyé et al. (2015) wrote a retrospective report following task performance, describing what was important to them when they made their decisions, and which things they paid attention to. As a surface exploration of these reports only showed high variability, they were not included in the earlier paper. This is a commonly observed phenomenon in cognitive science research. Verbal reports, because they are seen as too varied, complex, and inconsistent, are frequently discounted as unreliable introspective intuitions that cannot provide relevant insights into actual cognitive processes (Garner 1988). Here, we challenge this view by treating the data differently: not (only) as a direct resource to access participants’ conscious reasoning processes, but more importantly as linguistic data that allow for further access into cognitive focus, which may be unconscious. We combine qualitative (content) and quantitative (linguistic features) analysis using a methodological procedure called cognitive discourse analysis (CODA; Tenbrink 2015, 2020). This method analyzes verbal data to identify not only consciously verbalized thoughts, but also patterns in linguistic structure that highlight cognitive aspects of which speakers may not be aware. For current purposes, our specific interest lies in the participants’ cognitive focus when confronted with spatial uncertainty. We ask how information about direction sources as well as individual preferences for strategies affected participants’ cognitive focus, as reflected in their linguistic choices.

In the following, we explore three largely unrelated areas of research relevant to our current goals: how people follow route directions, how decisions are made under conditions of uncertainty, and how cognitive focus relates to verbalization of thought. We then introduce our experimental study and summarize relevant results previously reported in Brunyé et al. (2015), before addressing the new analyses of the retrospective reports in the present paper.

### Route directions

Verbal route directions have long been recognized as an important and revealing data source that highlights how people navigate spatial environments. They reflect crucial cognitive elements such as the sequential order of travel, the importance of choice points such as street intersections, the orientation toward landmarks, and the qualitative and vague way in which distance information is conveyed in temporal or spatial terms: *First, walk straight ahead until the second crossing, turn left, walk for a couple of minutes until you reach the church…* Such directions are typically accompanied by gestures supporting the imagined walking direction, as in *turn left* accompanied by a hand wave to the left. All of this seems natural to us, and indeed, these elements of route directions are well established as a conventional part of our cultural repertory. They correspond to the information needed for successful route navigation (Daniel and Denis 1998; Denis et al. 1997)—as opposed to additional, more individual types of information such as references to beautiful scenery, opportunities to buy groceries, or the likelihood of heavy traffic, all of which people consider in choosing routes (Hölscher et al. 2011).

In different parts of the world, directions might sound very different. People might orient more toward ubiquitously available environmental features such as *uphill and downhill* (Brown and Levinson 1993), or they might rely on gestures that represent the actual direction of a place, rather than the egocentric orientation of the traveler (Le Guen 2011). The fact that route directions are necessarily embedded in a cultural context is decisive for current purposes. For instance, the intuition that automatic GPS systems can be better trusted to provide turn direction information than to refer reliably to landmarks (Brunyé et al. 2015) must be related to culturally embedded knowledge about automatic devices’ capabilities (Rip 1989). This would be true independent of whether or not such knowledge is actually prominent in a wayfinder’s mind; much of it will be activated quickly and efficiently, without requiring substantial cognitive effort. However, it might be related to a distinct kind of cognitive focus, where subtle cues in a scenario guide attention toward specific aspects and away from others. Lepsien and Nobre (2006) demonstrated that cues can guide focus of attention on both a working memory and perceptual level. In our scenario, the cultural knowledge associated with the direction source information might serve as a cue, making particular aspects of a direction source’s reliability prominent.

Turn direction information is important in route directions for obvious reasons—the wayfinder needs to know which direction to turn at every choice point. In addition, knowledge of the general goal direction is a decisive cognitive factor during navigation (Dalton 2003; Hochmair and Frank 2000), especially when explicit planning is not required (Hölscher et al. 2011). This might make turn direction information particularly prominent, as specific turns will contribute to the wayfinder’s knowledge about, and orientation toward, the ultimate goal. Landmark information, in contrast, does not add to the wayfinder’s knowledge (or *sense of direction*) directly. Instead, landmarks anchor one’s position in the environment, identifying specific locations rather than general directions. Both aspects—anchored localization and goal-directed orientation—combine in the case of visible distant landmarks, such as a salient tower located close to a navigation goal. However, such a coincidence may not happen often; more frequently, landmarks during wayfinding are associated with specific parts of the route (Lovelace et al. 1999). Since knowledge about the general goal direction is decisive, it is not surprising that people rate turn directions as the most important element of route directions, followed by landmarks (Burns 1997). Also, unreliable landmarks can reinforce direction-based navigation strategies (Foo et al. 2005).

### Decision making under conditions of uncertainty

While research extensively examined how humans navigate known environments and follow accurate route directions, surprisingly little is known about navigation strategies when uncertain. However, it is actually fairly common for route directions to be less than entirely accurate. Reliable and complete route directions presuppose complete and accurate knowledge of the environment, which cannot be assumed even under the best of circumstances. Humans remember what is relevant for themselves, and this might mean that information relevant for the stranger asking for directions is not readily available (Tenbrink 2012). Moreover, mental representations tend to be distorted (Stevens and Coupe 1978; Tversky 1992), humans frequently confuse left and right (Storfer 1995), and their memory of landmarks and other spatial features might be outdated. In short, there are plenty of reasons why route directions may be incomplete or faulty. In line with this, a route giver’s uncertainty may be reflected in the way route directions are formulated (Tenbrink et al. 2011).

In addition, route directions are often given orally in advance, with no possibility for confirming successful communication when enacting the directions. In addition to having to deal with possible misunderstandings, the wayfinder is confronted with a considerable memory load. Since there is no way of visualizing the entire route based on the sparse spatial detail usually provided in route directions, the wayfinder would have to memorize these directions in their entirety. This may be virtually impossible; it is a well-established fact that retention of original wording fades quickly once comprehension has been completed (Sachs 1967), and that working memory faces substantial loads when people attempt to memorize a sequence of route instructions (Hund 2016).

Taken together, all of these factors combine to explain why people frequently get lost in spatial environments, despite having been given good instructions. And frequently, as a consequence of the combined challenges, wayfinders are confronted with situations where the remembered route directions do not appear to match the environment. They might remember that they have to turn left at the church, but find that there’s only a right turn available at the church, or that the only left turn is at a bakery. How decisions are made in these very common situations is surprisingly unclear from the available wayfinding literature. Our previous study (Brunyé et al. 2015) set out to explore people’s intuitive preferences and identified striking patterns for decision making under uncertainty that pointed to a strong influence of the direction source. However, the underlying cognitive processes that led to these results remained unresolved.

What influences people’s decisions under other kinds of circumstances, and what goes on in people’s minds as they reach a decision? Tversky and Kahneman (1974) suggested that humans have a tendency to use a limited number of heuristics, leading to quick decisions and judgments rather than taking the entire complexity of available factors into account. According to Todd and Gigerenzer (2000), it is precisely this capacity of simplifying situational and cognitive complexity that enables humans to make smart decisions efficiently and successfully, especially when confronted with the (rather common) situation of having to deal with too many uncertainties to be able to account for all eventualities. If it is impossible or highly demanding to determine the objectively optimal solution on a rational basis, it is useful to be able to draw on a limited number of cognitive heuristics to guide action. In the spatial domain, heuristics that have been identified to account for route choices include a preference for straight lines toward the goal direction with as few turns as possible (Dalton 2003; Hölscher et al. 2011; Sadalla and Staplin 1980), routes that head generally south rather than north (Brunyé et al. 2010, 2012), and routes with a particularly long stretch at the beginning (Bailenson et al. 2000). Even distances are estimated on the basis of heuristics (Hirtle and Mascolo 1991), and overarching navigation strategies are guided by spatial regions and conceptions of encompassing trajectories (Wiener et al. 2004). Altogether, there is clear evidence for cognitive shortcuts under different navigation circumstances, explaining humans’ ability to act even when hampered by uncertainty (Tenbrink et al. 2011).

If decisions are guided by a limited set of heuristics, what guides people’s reliance on a particular heuristic? One possibility is that individual differences generally account for strategic preferences, such as relying on landmarks as opposed to global directions (Lawton 1996; Pazzaglia and De Beni 2001; Shelton and McNamara 2004). However, even strong personal preferences will be affected by situational factors (Lipshitz and Strauss 1997; Maule and Svenson 1993), as confirmed by our previous study (Brunyé et al. 2015): strategies shifted from an even distribution between landmark vs. turn-based choices to a clear preference for turn direction information from automatic devices, and a clear preference for landmark information when the direction source was a clearly identified human being.

### Cognitive focus and verbalization of thought

Cognitive shortcuts, such as decision heuristics, have the advantage of simplifying complexity, and guiding rational considerations within manageable limits (Todd and Gigerenzer 2000). Inevitably, such a strategy will guide a person toward just those aspects of the situation that are relevant for applying the heuristics in question, in line with the basic conception of attention itself—as defined ingeniously by William James more than a century ago (James 1890):

It is taking possession of the mind, in clear and vivid form, of one out of what seems several simultaneously possible objects or trains of thought. Focalization and concentration of consciousness are of its essence. It implies a withdrawal from some things in order to deal effectively with others.

Thus, a landmark-based navigation heuristic will guide attention away from other spatial information and center primarily on landmarks, just as a region-based strategy will mean that humans pay particular attention to existing regions in the environment (Wiener et al. 2004). Such attentional biases are regularly represented in language (Marchetti et al. 2015). For instance, Talmy (2007) summarized many ways in which linguistic features reflect cognitive attention processes, such as foregrounding particular aspects in sentence structure, lexical complexity (compare *One of my parents’ sisters* to *One of my aunts*), and the relative salience of Figures and Grounds (compare *The bike is next to the house* to *The house is next to the bike*). Furthermore, Carstensen (2015) demonstrated how spatial terms express perspectivations of space in various ways, related to mental operations of selective attention, preconceptions, and perceptions of space. For instance, the use of *over* and *under* is closely linked to visual experience of orthogonality to a horizontal line or plane.

Using linguistic devices such as these, attention is subtly represented by the way people formulate their utterances, including which aspects they choose to express at all (Talmy 2000). For instance, theoretically, any movement from a place to another will contain a starting point, a trajectory, and an endpoint; however, speakers typically express just one of these: *He left the house* vs. *She took the scenic route* vs. *They arrived at the school.* Each of these clearly signals attention to just one aspect of the route—the one that is relevant in a current communication context. In a specific discourse context, a preference across speakers to refer to starting points (such as *the house*) more than trajectories (such as *the scenic route*) or end points (such as *the school*) therefore reveals their attention focus on the starting point, which in turn depends on the context of language production (Tenbrink 2015). Such a preference would naturally coincide with a preference for verbs that relate to the starting point (such as *leave*) over those that relate to the end point (such as *arrive*).

Verbalizations associated with specific thought processes can therefore reliably reflect cognitive focus, not only on the basis of *what* people happen to mention in a verbal protocol (Ericsson and Simon 1984), but also specifically by making use of this intricate capacity of language to represent attention (Talmy 2007). Analyzing the associated linguistic phenomena in detail can reveal the attentional focus of speakers (Tenbrink 2020) in a way similar to detecting manipulation of readers’ attention in news discourse (Alharbi and Bahmani 2015), based on the insight that readers’ attention is guided by the choice of particular expressions (Marchetti 2010). In this study, we set out to examine whether differential cognitive focus was associated with the decisions made by participants under conditions of uncertainty, as expressed in language, using analysis criteria tailored to our specific scenario.

## The experiment

As reported in Brunyé et al. (2015), we confronted participants with the dilemma of conflicting landmark and turn information. After reading a sentence such as *turn right at the pharmacy*, they were shown a situation in which they saw a pharmacy associated with a left turn and a right turn with a different landmark. This allowed us to directly differentiate participants’ priorities when making their decisions. We expected that individual differences as well as information sources would affect these strategies in systematic ways. In three conditions, we tested this prediction as follows. Participants read route directions such as *To get to the metro station, take a right at the Pharmacy* toward a goal location in an unfamiliar environment. The environment was then shown as an abstract depiction consisting of a straight path and two short intersecting roads to the left- and right-hand side, respectively. At the end of each intersecting road was a symbol that indicated possible goal locations, and participants were tasked to select the goal location they believed was correct. In many cases (serving as fillers), the route directions given to the participants accurately corresponded to the depicted environment, leaving no dilemma but only a simple route direction following task. In the experimental trials, the environment posed a dilemma and participants’ decisions could differentiate reliance on either landmarks or turn directions.

Three conditions differed only with respect to the source of route directions. In Brunyé et al. (2015) these were presented as individual experiments, but since the participants were taken from the same population they can be equally represented as between-subject conditions, which makes more sense for current purposes (cf. analyses spelled out below). In the control condition, participants did not receive any specific information about the direction source. The GPS condition contained information about an automatic GPS device that was either high or low in reliability. In the human condition, instructions elaborated on a human route giver who was described as either highly familiar or relatively unfamiliar with the environment. In the following, we present details of each condition along with a brief account of the patterns of behavioral results previously found across the three conditions (experiments), as reported in Brunyé et al. (2015), before moving on to present the new analysis of retrospective verbal reports. In particular, we will examine what participants reported to be important for their decisions, as well as systematically identifying linguistic features that highlight cognitive focus associated with the behavioral results.

### Conditions

All three conditions addressed participants' strategies when confronted with a route direction that conflicted with the perceived environment. Participants read a brief route instruction and were then shown a schematic top-down view of a spatial scene containing street intersections and landmarks, on the basis of which they made a decision of where to go. After 64 tasks of this kind, participants were asked to describe how they solved the task in general terms and what they paid attention to, thinking back about how they did it.

In the control condition, participants were not given any particular information about the route source. This condition (Experiment 1 in Brunyé et al. 2015) explored individual differences in spatial strategy adaptation under conditions of uncertainty. We expected that participants’ decisions could be predicted by their personal preferences for either landmark- or turn-based information.

The GPS condition (Experiment 2 in Brunyé et al. 2015) was designed to test whether the strategies identified previously would carry over to a scenario where the route information was given by a source that is not normally associated with landmark information—an automatic GPS device. In line with current attempts to integrate such information into current systems (e.g., Burnett 2000; Hile et al. 2008), we further manipulated the assumed degree of reliability by either stating, in half of the cases, that the route was provided by a software that is “a first version of a new GPS device and is untested for accuracy and details,” and in the other half, that it is “the 4th version of a popular GPS device that has proven accuracy and details.” There were two versions of the low-reliability and two versions of the high-reliability devices, represented by distinct fictitious brands and logos.

In the human condition (Experiment 3 in Brunyé et al. 2015), we asked whether human route givers might be associated with a different kind of fallibility than automatic devices. To ensure that participants conceptualized the direction source as a real human being, we provided them with a short description that included a gender-specific name (such as Michael or Sarah). The description also differentiated between knowledgeable and less knowledgeable route givers by detailing that they were, in half of the cases, “visiting” and had only been in town “for a couple of days,” or alternatively “born and raised in the town.”

### Participants

One hundred and eighty Tufts University students (81 male; mean age 19.8 years; 60 in each condition) participated for monetary compensation. All signed informed written consent in accordance with the Tufts University Institutional Review Board guidelines.

### Materials

In each condition, 64 sentences described the way from a starting location to a final destination by reference to a landmark (a common salient location, such as a theater or hospital) at a decision point, as well as a turn direction (i.e., left or right). Half of the route directions included a left turn and the other half a right turn. The syntax was counter-balanced between the options a) *To get to the hospital, take a left at the theater*, b) *To get to the hospital, at the theater take a left*, c) *Take a left at the theater to get to the hospital*, and d) *At the theater, take a left to get to the hospital*. Follow-up analyses confirmed that these syntactic options had no effect on the results.

**Fig. 1:**
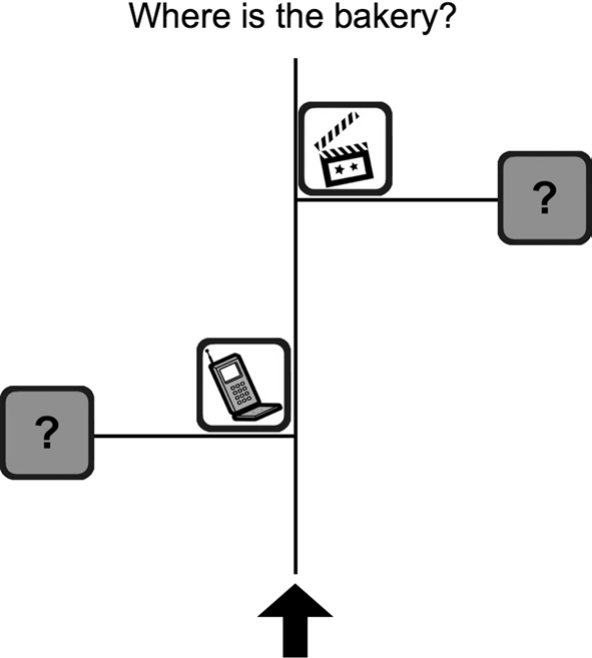
Example map stimulus

Each route direction was paired with one of 64 schematic maps (see Fig. 1 for an example). Each map showed a black arrow at the bottom that indicated the orientation from the starting point, along with a street running vertically upward from it. Further upward, two streets intersected with the vertical one, one to the left and the other to the right. In half of the maps, the one to the left came first, and in the other half, the one to the right. Each of these intersections had a landmark adjacent to it, represented with grayscale icons from Microsoft Office clipart framed by black rectangles. These were located either below or above, and to the left or right of, the intersections. Finally, a possible destination was shown at the end of each intersecting street, represented by a salient question mark in a black rectangle.

Critically, on half of the 64 trials, the information given in the route directions was consistent with the map (*non-dilemma* trials), and on the other half, it was inconsistent (*dilemma* trials). For instance, an instruction could say: “To get to the bakery, take a right at the cell phone store”. However, in a dilemma trial, the cell phone store (the decision-point landmark) would be adjacent to a left turn, whereas there would be an option to turn right at another landmark not mentioned in the instruction (e.g., a cinema). In half of the dilemma trials, the decision-point landmark was at the first intersection, and in the other half, it was at the second intersection. Participants indicated their choice by mouse-clicking on one of the two question marks indicating a possible destination. In dilemma trials, their answer would be consistent with either the landmark information (e.g., taking a left turn at the *cell phone store*) or the turn information (e.g., taking a *right* turn at the cinema). In non-dilemma trials, their answers would be consistent with both parts of the associated route instruction, unless they made an error.

### Procedure

After providing informed consent and reading task instructions, participants performed a practice trial and then completed the main experiment. The stimuli were 64 trials grouped in four blocks of 16 trials in random order, presented on 24” monitors using SuperLab® software (v4.5; Cedrus, San Pedro, CA, USA). In each trial, participants read a route direction consisting of a single sentence and clicked the mouse when they were ready. The next screen focused their visual attention to the monitor's center by showing a grey box labeled “GO,” which participants had to click. This also served to position the cursor starting position into the middle of the screen. The next screen showed a map with two possible locations to choose from (Fig. 1). Participants selected a destination by clicking on one of the two question marks, which were located equidistant from the monitor's center. Their choices and response times were recorded, and the next trial started directly following the participant's response.

In the control condition, the four blocks of 16 trials did not correspond to any manipulation in the experimental design; the blocks were simply included for consistency and to allow for a short break between blocks. In the GPS condition, the four blocks corresponded to the four different GPS devices (two low-reliability and two high-reliability brands). In the human condition, the four blocks corresponded to two female and two male human route givers, in each case with one of them highly knowledgeable and the other not very knowledgeable of the environment.

The critical procedure for the current work involved the retrospective reports (not previously reported as deemed irrelevant for analysis in Brunyé et al. 2015). After completing the 64 trials, participants viewed the following retrospective question on the computer screen: “Please describe how you solved this task in general terms. What was important to you when you made your decisions? Which things did you pay attention to? Please report everything that comes to your mind when thinking back about how you did this task.” Participants typed their retrospective report. Following this free response task, they were asked a range of more specific questions about the task, including a direct question about their preferences (landmark or turn information). They also filled in further questionnaires concerning their general spatial strategies, preferences, and representations (see Brunyé et al. 2015 for details), as well as demographics.

### Behavioral results

As reported in detail in Brunyé et al. (2015), participants were highly accurate in non-dilemma trials across all conditions, with average accuracy close to 100% across the board. Non-dilemma trials were also consistently solved more quickly than dilemma trials, by > 1.5 s on average. In dilemma trials, most participants used one of two possible strategies with considerable consistency, going either with the landmark or with the turn information; patterns differed across conditions as reported below. In all conditions, some participants switched frequently between both possibilities. In the control condition only, they overwhelmingly tended to choose the second intersection shown in the map; this pattern disappeared in the GPS and human conditions. Across all three conditions, strategy choice could not be traced back to any of the general spatial preferences and strategies as assessed by the questionnaires administered in this study. Also, strategy choice did not appear to develop in any consistent way across time. In the control condition only, males relied on landmarks more, whereas females preferred turns, which seems to be at odds with previous findings pointing to the reverse pattern (Lawton 1994; Sandstrom et al. 1998). This pattern, as well, disappeared in the GPS and human conditions. Strategy choice appeared to be highly conscious, as participants’ subsequent subjective assessment of reliance on landmark versus turn information, elicited directly in a questionnaire, reliably corresponded to their actual performance across all three conditions.

A direct comparison between the three conditions (control, GPS, and human) shows that direction source systematically influenced strategy scores. In the control condition, preferences were fairly evenly distributed between consistent reliance on either landmark or turn information, and a somewhat lower number of participants who switched between the two. In the GPS condition, participants’ decisions were clearly skewed toward turn direction information, and in the human condition, the majority of participants showed a landmark-based preference. Further details will be provided below, as we introduce a categorical classification of participants’ preferences for the purposes of systematic linguistic analysis, which we address next.

### Analysis of retrospective reports

Participants provided retrospective thoughts on their approach to the main experiment before scoring their preferences for landmark or turn information in answer to a direct question. Here we analyze these retrospective reports with the aim of identifying relevant conscious cognitive processes of decision making, as well as linguistically represented patterns of cognitive focus that shed further light on the (possibly unconscious) mechanisms underlying participants’ conscious strategies. For this purpose, we followed the procedures of cognitive discourse analysis (CODA, Tenbrink 2015, 2020). This included explorative content analysis to provide a sense for explicitly verbalized strategies beyond the mention of landmark vs. turn preference, followed by systematic analysis of decisive linguistic elements that highlight cognitive focus in ways that participants may not have been aware of and accordingly did not report explicitly.

In order to get a clearer sense of the cognitive processes expressed in retrospective reports in relation to different preferences, we classified participants according to their behavioral results. In the strategy score reported in Brunyé et al. (2015), a perfectly consistent behavioral choice pattern is represented as − 1 for landmark preference and 1 for turn preference, whereas a pattern that would allow for turn and landmark choices to equal degree would score 0. Based on content inspection (see below), it appeared that participants either aimed at consistency across trials in choosing either landmark or turn information, or consciously switched strategies under certain circumstances. A threshold halfway (0.5 or − 0.5) between consistent strategies (1 or − 1) and equal distribution (0) seemed a sensible criterion for distinguishing between these strategies. Using this criterion, we classed individual responses as landmark, switchers, or turn, as represented in Table 1. While the number of switchers remained relatively constant across the three conditions, the number of participants using a consistent landmark- or turn-based strategy was almost equal in the control condition, but changed to overwhelming landmark preference in the human condition along with enhanced turn-based preference in the GPS condition.

**Table 1:** Numbers of participants across conditions who preferred landmark or turn information, or switched between them, according to behavioral results

Condition/category	Control	GPS	Human
Landmark	22 (+ 1) = 38.3%	15 = 25%	40 = 66.7%
Switcher	15 (+ 1) = 26.7%	18 = 30%	16 (+ 1) = 28.3%
Turn	21 = 35%	24 (+ 3) = 45%	3 = 5%

Numbers in brackets indicate additional participants who failed to provide retrospective reports. Each condition included 60 participants

**Fig. 2:**
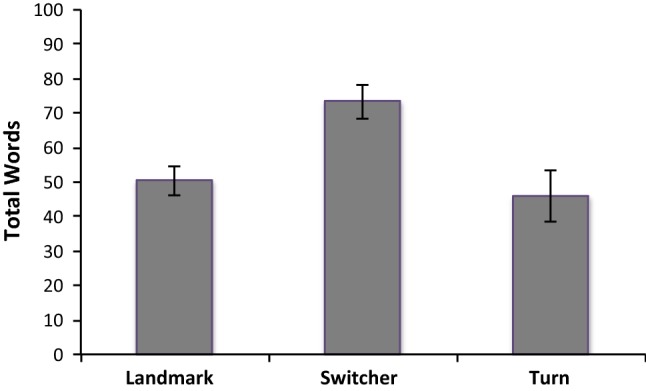
Word count across all conditions according to preferences

**Fig. 3:**
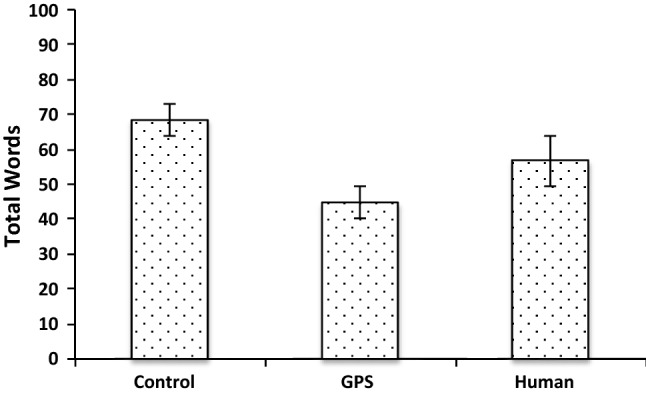
Word count across preferences according to condition

Switchers wrote longer statements containing more intricate considerations than those with consistent landmark- or turn-based strategies. This was consistent across all three conditions, *F*(2, 165) = 7.72, *p* = 0.001, MSE = 1182.79 (see Fig. 2). There was no interaction with condition, but a main effect of condition (*p* = 0.002), where participants produced fewer words in the GPS condition (Fig. 3).

### Exploratory content analysis

Many participants explicitly reported decision-making strategies in their retrospective report, even before they were directly asked about landmark or turn information preferences. In particular, they frequently reported the ways in which they dealt with dilemma trials, highlighting their general focus of attention during their decision-making processes. Consistent with the questionnaire results reported above, in all three conditions speakers overwhelmingly reported strategies that corresponded to their behavioral patterns. For instance, in the control condition, a participant who systematically followed landmark rather than turn information reported:

1. *The important piece of information was the type of building near the turn. I feel like it is easier to remember what kind of landmark to turn on rather than what direction you turn in.*

And a participant who systematically followed turn information wrote:

2. *I paid more attention to whether the route directions said to go to the left or right, than I did to the pictures.*

Thus, consistent behavioral strategies typically came with correspondingly clear-cut retrospective statements that pointed to a simple heuristic that participants had used. Participants often gave explicit reasons for these choices that entailed imagining a realistic wayfinding situation. For example, in the neutral control condition, people who preferred landmark information expected that route givers are more likely to confuse left and right than landmarks, and that landmarks stick in memory as they are prominent and important, in contrast to turns which are unstable, i.e., perspective-dependent; the direction changes if you come from somewhere else. Participants who relied on turns often attended to the exact location of the landmark and argued that route givers may misremember its relation to the turn, or they may choose to refer to it even if it is a little distant from the correct turn. These indicative examples of explicit reasoning already provide a flavor of the cognitive focus adopted by participants, associated with different strategies. Participants were actually aware of their selective attention processes, and frequently referred to this explicitly, as in the above examples. Typical linguistic indicators in this respect that frequently re-occur in the retrospective reports are *attention*, *important, focus* and the like.

A relatively simple explanation motivating switching behavior in the control condition is:

3. *At first I paid more attention to what I was supposed to be turning at and not the direction, but afterward I paid more attention to which direction I was supposed to go.*

However, various people in the same condition reported rather complex decision-making strategies, such as the following examples:

4. *Usually the landmark was more important than the general direction. If the landmark, and the direction at the landmark did not work, I would see if it was possible to take that direction turn further ahead and the person giving directions may think of the street that goes in that direction near the landmark, instead of right next to the landmark.*
5. *I had to look for the place that was given to in the directions and then locate if there was a destination in the directions near it. If the directions didn't easily point to a location, I would pretend I was standing outside of the building and then see in which direction the directions told me to go. I then proceeded to pick the destination from there.*

Participants also reasoned that order or distance was decisive. They would decide on the turn or landmark based on whether the correct landmark came first, or based on the distance between the landmark and the turn (which depended on the landmark placement). This suggests that switchers were not simply inconsistent in their responses, but invested considerable effort toward producing reasonable answers, paying attention to various scenario aspects including sequential order and relative distance. This is even true for a participant who admitted to guessing:

6. *If it was clear which way to go, there was no problem. If it was unclear I just guessed. I was thinking about how bad I am with directions and how when I get lost when following directions, I look at the streets and the shops there and go off of a vaguely intuitive sense of what seems right. Obviously that was a lot harder to do in this experiment but I tried it anyway.*

In the other conditions, general content patterns were similar, though participants had clearer tendencies to argue for turn-based (GPS) or landmark (human condition) strategies, corresponding to the behavioral results. For instance, a participant who preferred turn information in the GPS condition wrote:

7. *I paid the most attention to whether or not they told me to turn left or right since that is probably more accurate than a GPS's knowledge of where local businesses are. So I chose based on right and left directions.*

In contrast, a participant who relied on landmarks in the human condition wrote:

8. *I figured that they would remember the landmark rather than the direction if they didn't correspond.*

Some participants decided based on the information source’s reliability, such as the following switchers in the GPS and human condition, respectively:

9. *The proven accuracy of the GPS system was the most important factor in my decision. For the beta versions 1.0 of the GPS devices I figured that the directional location (left/right) was more important than the location of the other building. For the GPS devices with a proven track record I trusted that they knew what they were doing so even if the initial direction was incorrect I figured that it might have known something about the town maybe a no left turn or something.*
10. *How long the person has been in town and how old they sound by the description. For visitors, I used landmarks more than directions and for residents, I used a combination of distance between intersections and landmarks.*

These examples shall suffice to provide some initial insights into the considerable individual differences in reasoning processes between participants, interacting with the different conditions that gave participants a clue as to what aspects to give preference to. In order to probe deeper into the effects of cognitive focus on decisions under uncertainty, a more systematic analysis of linguistic features in the reports is required, to which we turn next.

### Systematic linguistic analysis: procedures

Explicit content of the retrospective reports indicated that participants were aware of paying attention to either landmark or turn information, plus some additional factors, based on their conscious reasoning processes. We now aimed to identify aspects of the verbalizations that reflected cognitive focus beyond explicit mention and thus possibly without participants’ conscious awareness. This analysis was done by identifying specific linguistic indicators of cognitive focus across a range of categories as explained below.

We used Excel software facilities to count these indicators. The decision to rely entirely on semiautomatic procedures rather than intricate manual annotation was motivated in three ways. First, item-by-item manual annotation is notoriously time-consuming and impractical for large data sets. While it might have been feasible for our specific purposes, replicability and wider generalizability of our study would be hampered if manual annotation procedures were necessary. Second, manual annotation is prone to human error and both intra- and inter-coder inconsistency. By identifying predictions that could be operationalized toward automatic analysis, the important danger of subjectivity was avoided. Third, the identification of linguistic markers for specific cognitive processes is useful for future purposes well beyond our current aims, such as the automatic detection of discourse properties and other types of diagnostics in cognitive science.

To start addressing linguistic indicators of cognitive focus, we identified references to elements that were central to the situation. The conceptual configuration that contributes to the decision about landmark or turn information consists of three decisive elements: the *participants* themselves, the direction source (the *route giver*), and the visually presented *scenario*. Participants could focus their attention on one of these, leading to a bias in cognitive focus that we hypothesized might be associated in various ways with conditions and strategy differences. These would not be expressed explicitly in content but rather be reflected by frequency of key reference terms. For instance, if there is little information about the direction source, participants would be more likely to focus on either themselves or the scenario, leading to the behavioral results we identified as described above. To address the linguistic expression of such patterns in cognitive focus, we identified references to the self (I-Ref), or to the route giver (R-Ref), or to the scenario (S-Ref).

For the I-Ref category, we simply counted all occurrences of the first-person pronoun *I,* including *me* and *my.* R-Refs were more complex as the route giver could be referred to in more than one way, including implicit and elliptical phrases. We counted all occurrences of terms that (upon inspection) were used recurrently in connection with the direction source, namely: *giver, person, giving, system, GPS, they, people, he, instructor, source, who, gave, individual,* and *their*. There were no occurrences of “she” across the whole study, and the pronoun “it” was rarely if ever used to refer to the direction source (but frequently with other meanings). Notice that this list excludes neutral references to the *instructions* or *directions* given to the participant; references in this neutral form would not specifically reveal a cognitive focus on the route giver. This is a meaningful (though potentially subconscious) difference when choosing linguistic terms to describe cognitive processes, as references to *instructions* reflect a focus on the product instead of the producer.

The category S-Ref included references to terms reflecting concrete aspects of the scenario (excluding the key terms participants needed to express their choices: *landmark*, *turn*, and *direction*, as well as generic or abstract terms like *picture*, or *symbol*), namely: *shop, store, street, station, intersection, house, road, supermarket, pharmacy, gym, church, town, library,* and *building*. All of these reflect awareness of specific scenario aspects beyond the actual choices made.

Next, we investigated the reflection of cognitive focus in verb use. Halliday and Matthiessen (2014) highlight the central function of *processes* for discourse organization. The main process type in each clause affects the entire structure of the clause in systematic ways. The most diverse category of processes is called *doing* (or *material*) processes such as *give, put, make* etc.; these presuppose the presence of an *actor* along with other participants such as *goals* and *beneficiaries* of the *doing* process. For instance, in *Jason gave the boat to his friend, Jason* is the *actor, the boat* is the *goal* of the *giving* process, and *his friend* is the *beneficiary. Relational* processes are typically forms of *be* as the clause’s main verb. In these cases, participants are identified as (or related to) something, as in *Jill is the instructor*, or *John is tall. Mental* processes represent cognitive processes, such as *think, consider, decide* etc., which imply a *senser* and a *phenomenon*.

The last two process types are particularly relevant for current purposes. As outlined by Halliday and Matthiessen (2014) and elsewhere in the tradition of systemic functional grammar (SFG), relational processes are generally found in abstract discourse that represents the world with a focus on facts, identities, and relationships, rather than actions or thoughts. This would seem to be associated with a cognitive focus on the scenario rather than the direction source or the participants’ own thoughts. In contrast, the direct representation of mental processes should correspond to a cognitive focus on either the self (with the participant describing their thoughts), or the direction source and their possible thoughts. We therefore decided to analyze relational and mental processes in our data. No other verbal process category (e.g., material) was analyzed as no specific cognitive focus could be expected to be associated with them.

To address the possible relationship between mental or relational processes with cognitive focus, we introduced an operationalization that incorporated the idea (central to SFG) that basic functions can be represented in more than one form. We inspected the data to identify recurring verbs and verb phrases as linguistic markers that represent either mental or relational processes, in order to enable automatic counting. In our operationalization, the mental process category contained the terms *attention, figured, ignore, focus, thought, look, confuse, disregard, decide, realize, mind, important, concern*. While not all of these terms are verbs (or can be nouns depending on context), critically all of them reflect a focus on the participants' thought processes in the contexts in which they are used. For instance, *attention* frequently occurred in the phrase *pay attention*, in various grammatical forms.

Forms of *be* (which, in our context, mostly occurred as *was, were,* and *is*) can serve the function of a main relational process as explained above, as in “the location *was* nearby.” This corresponds to our primary target for analysis, namely, a focus on the scenario. However, when identifying these terms in the data, we noted that these same forms also frequently served as auxiliary verbs in passive constructions involving a different type of process, as in “I focused on the landmark that *was* said not the directions that *were* given because often times the rights and lefts *were* confused.” According to SFG (Halliday and Matthiessen 2014), one main function of the passive voice is to eliminate information about the agent of an action. For instance, in the given example, the direction source is left implicit, rendering the description more abstract and underspecified (with respect to the route giver) than an active clause would have been. In effect, then, the passive voice serves a similar purpose as the use of relational processes in our context: a de-emphasis of the direction source, a cognitive focus on other aspects of the scenario. Therefore, we searched for markers of relational *and* (co-incidentally) passive clauses, using the already identified markers *was, were, is.*

**Fig. 4:**
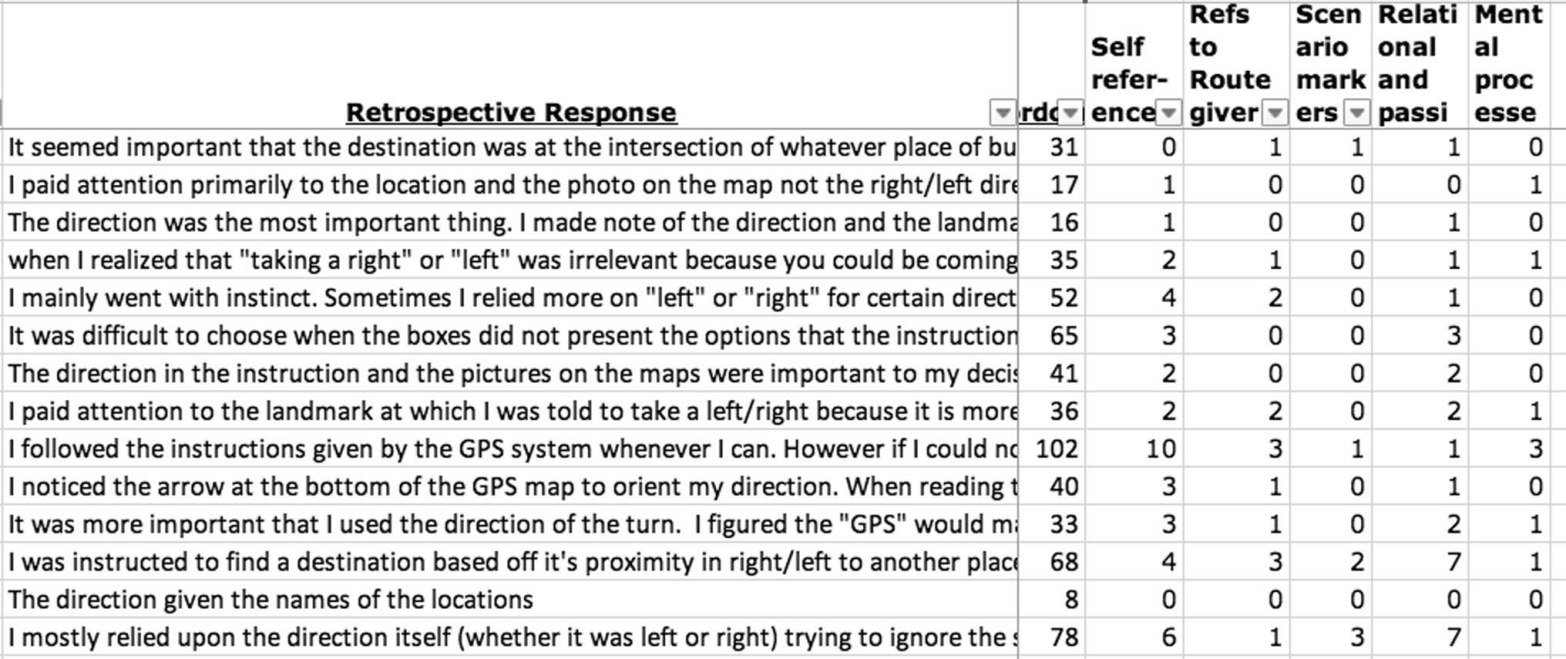
Screenshot of annotation in the “GPS” condition. The second column (after the response) provides the word count and the following columns count the linguistic items that the software detected in the response

All our categories were defined and iteratively checked, ensuring that they captured the intended linguistic items. Figure 4 illustrates our semiautomatic annotation in Excel.

### Systematic linguistic analysis: results

Results are reported as percentages of all markers counted in this study in the way just outlined. This counteracts possible biases contributed by verbosity or by content of a kind not focused on in our analysis. We addressed the patterns of linguistic markers revealing cognitive focus (as dependent variable) against two independent variables for the present study: the effects of condition (i.e., whether the direction source was not in focus, or a GPS system, or a specifically described human being); and the effects of strategy choice (a focus on landmarks or on turn information, or switching between them). Note that strategy choice was a dependent variable in Brunyé et al. (2015), as our original research addressed the effects of condition on strategy choice. Here, in contrast, we address the effects of specific strategy choice decisions, under different conditions, on linguistic representations of cognitive focus.

**Fig. 5:**
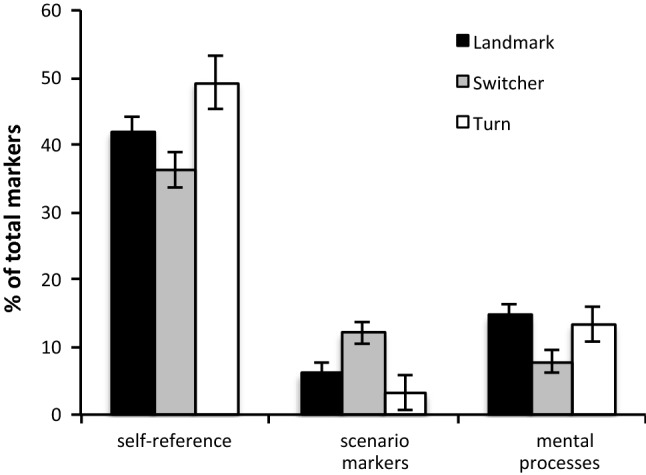
Main effects of strategy preference for relative frequency of linguistic markers (where significant)

We conducted univariate analysis of variance (ANOVA) with between-participant factors of direction source (control, GPS, human) and strategy preference (landmark, switch, turn) for all measures. We report first the main effects and later interactions between the variables. The analysis revealed a main effect of strategy preference for the use of I-Refs (*F*(2, 163) = 3.402, *p* = 0.036, MSE = 367.36) and S-Refs (*F*(2, 163) = 5.706, *p* = 0.004, MSE = 135.83). Across conditions, participants who relied on a turn-based strategy used relatively more I-Refs (*I, me,* and *my*) but fewer S-Refs (Fig. 5) than people relying on other types of strategies. In contrast, people who switched their choices according to features of the scenario showed the opposite pattern. Also, references to mental processes were least frequent in switchers. No other main effects of strategy choice were found.

**Fig. 6:**
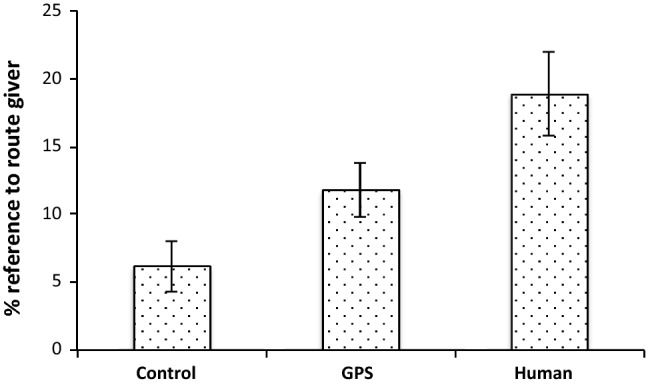
Main effect of condition in use of references to route giver

We found a main effect of condition for R-Refs (*F*(2, 163) = 6.46, *p* = 0.002, MSE = 204.46, see Fig. 6). As expected, participants in the control condition did not frequently focus on the route giver. After all, no details were given concerning the source of the route instructions. The highest relative frequency of route giver references occurred in the human condition, where details about a human route giver were given. The GPS condition was intermediate in this respect. No further main effects of condition were found.

**Fig. 7:**
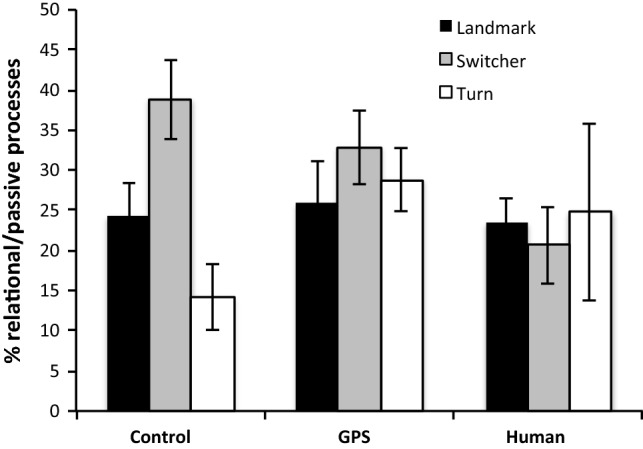
Relative frequency of markers indicating relational/passive processes according to strategy choice and condition

In terms of interactions, the ANOVAs revealed a marginal interaction (*F*(4, 163) = 2.36, *p* = 0.056, MSE = 365.83) between condition and strategy choice in the use of relational/passive processes, as indicated by the use of *was, were,* and *is.* In the control condition, differences according to strategy choices were most pronounced, with switchers relying on relational/passive processes far more frequently than people with clear strategies. This corresponds to their focus on the scenario rather than the self. This difference disappeared with the other two conditions in which other types of effects and preferences emerged (Fig. 7).

**Fig. 8:**
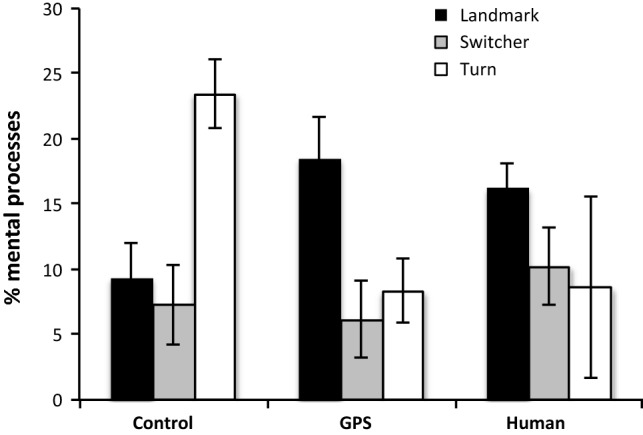
Relative frequency of markers indicating mental processes according to strategy choice and condition

Likewise, strategy choice was related to the use of mental processes (*F*(2, 163) = 4.55, *p* = 012, MSE = 145.12), again most pronounced in the control condition, as supported by an interaction between condition and strategy choice (*F*(4, 163) = 5.53, *p* = 0.000). These patterns are somewhat more intricate (Fig. 8). Mental processes were most frequently used by people relying on turns in the control condition, but by people relying on landmarks in the two other conditions. Switchers generally referred to mental processes to a lesser extent, corresponding to their greater focus on the scenario. Recall that since landmarks are generally highly preferred in the “human” condition, there were only three people in the turn/human category, explaining the high variance. No further interactions were found.

## Discussion

We set out in this study to identify differences in cognitive focus that may shed light on the ways in which different direction sources affect how people interpret ambiguous route instructions. To address this, we identified patterns of linguistic markers in the retrospective reports that indicated cognitive focus given different strategical choices under different conditions.

Arguably the most predictable of our results concerns how information about the direction source affected the use of linguistic markers indicating a cognitive focus on the route giver. With limited information about the direction source, the retrospective reports had little evidence for a cognitive focus on the route giver (see Fig. 6). In contrast, when the instructions described a human route giver in detail, participants referred to route givers more extensively when explaining their own strategies during decision making. With a GPS as direction source, the references fell in between these two extremes. It could be argued that our annotation procedure may not have captured all the specific ways in which participants referred to the GPS. However, this seems unlikely as we closely inspected the data multiple times to avoid missing crucial reference forms, and also given the ease of referring to the *system* or *GPS* as opposed to the more varied ways in which speakers referred to a human route giver (e.g., *giver, person, instructor, individual* etc.). Assuming, therefore, that there is a genuine difference in cognitive focus on the direction source between the human and GPS conditions, this may be related to the fact that fellow humans are easier to identify with and relate to than automatic systems, which are understood to function fundamentally differently to humans (Madhavan and Wiegmann 2007), and which require specific experience for trust and relevant expectations to develop (Schaefer et al. 2016). This may also account for our finding that participants overall produced fewer words in the GPS condition than in the other conditions. As the participants were not used to this particular automatic system, they had less to say about it. It stands to reason that this kind of effect may change as personal virtual assistance systems become more common, and users increasingly relate to them in various ways (Purington et al 2017).

The general pattern underlying how information about the direction source influenced route giver references, however, is straightforward and uncontroversial. If the instruction given to the participant emphasizes specific features of the direction source, the strategy choices will be influenced by a greater cognitive focus on the route giver, and the retrospective reports will reflect this focus systematically. The fact that our annotation system captured this effect serves as a proof of concept, or validation of our approach: while neither surprising nor ground-breaking as such, this finding confirms that the linguistic analysis does what it is supposed to do. This is in line with earlier approaches to treating verbal protocols as data (Ericsson and Simon 1984), with a multitude of studies implementing this idea in various ways, including the specifically linguistic approach of *Cognitive Discourse Analysis* as elaborated in Tenbrink (2020). Notably, a mere scrutiny of content would not have been sufficient to confirm cognitive focus in our study, as the content of the retrospective reports proved to be extremely diverse. Rather than explicitly stating their cognitive focus, participants would naturally report their individual strategies for decision making, which were highly varied. However, their use of references to the route giver, as identified in our current study, revealed that they were consistently more focused on the route giver under conditions that made the direction source more prominent.

None of our other linguistic measures was directly dependent on the nature of the direction source, and there is no particular reason they should be. Ultimately, our main interest concerned the linguistic expression of strategies, both in general and under different conditions. Recall that participants could interpret ambiguous route descriptions either based on landmark information or on turn information. As reported in Brunyé et al. (2015), they either relied consistently on landmark or turn information, or they switched between the two options according to heuristics or criteria that they identified themselves. These strategies depended on condition, where receiving directions from a human led to a higher reliance on landmark information and directions from a GPS led to a higher reliance on turn information. Nevertheless, all strategies were used (to different degrees) in all conditions, allowing us to examine cognitive focus as reflected in linguistic choices given different strategical choices. In the following, we will first report how cognitive focus relates to strategies in general, starting with strategy switchers whose reports were most elaborate and systematically differed from non-switchers, and then examine how cognitive focus is associated with strategy choices related to different kinds of direction sources (conditions).

Our analysis of the retrospective reports revealed that participants who switched strategies wrote longer reports than those who did not (see Fig. 2); they clearly had more complex thoughts to report, and more intricate explanations for their decisions. While we did not directly address complexity or amount of detail in our linguistic data based on specific linguistic measures, word count is a suggestive indicator in this respect (Tenbrink 2020). Inspection of the linguistic data as well as indicative qualitative content analysis (reported above) confirms this for the present study, as participants who switched strategies frequently elaborated on the specific situations influencing them to switch.

Independent of word count, switchers differed from non-switchers in that their language revealed a greater focus on the scenario, and a relatively lower focus on themselves and their thoughts (Fig. 5). Switchers had lower counts of first-person personal pronoun (*I, me, my*) and linguistic markers of mental processes (such as *thought, decide, realize*), along with higher counts of terms referencing concrete aspects of the scenario (such as *shop, street, house*). This supports a cognitive focus on specific scenario features, in line with our observation that switchers’ higher word count reflected a more detailed elaboration on specific situations influencing them to switch. This is in contrast to more abstract considerations with a higher focus on the self in the non-switchers’ reports. This cognitive orientation (away from the self and toward specific scenario features) seems specific to switchers, rather than being influenced by the nature of the direction source.

Taken together, our findings on the switchers’ cognitive focus add to a host of previous studies on the procedures and efforts of switching between strategies, which comes with additional cognitive cost (e.g., Lemaire and Lecacheur 2010). The ability to switch between strategies is seen as beneficial and desirable in terms of cognitive flexibility (Schillemans et al. 2011), but the added cognitive challenge means, for instance, that switching strategies can become more difficult with age (Harris et al. 2012). In our context, switchers showed more attention to scenario details rather than fixating on their own concerns, and thereby demonstrated higher flexibility across specific trial situations—contrary to a consistent strategy of relying on one aspect only, which would have been easier and less costly to pursue. The various linguistic features of the retrospective reports consistently reflect their engagement with the individual tasks (trials).

The switchers’ focus contrasts with non-switchers, i.e., those selecting a consistent turn- or landmark-based strategy. Those pursuing a fairly consistent turn-based strategy exhibited a cognitive focus nearly opposite of the switchers; they referred more to themselves using first-person pronouns and only a few scenario markers. Clearly, with a focus on turn direction information, the specific details of the scenario mattered less overall, and the participants considered their own abstract thoughts more explicitly, using phrases such as *I paid more attention to…* People pursuing a landmark-based strategy were in between the turn-based and the switcher strategy. This may be related to the fact that landmarks were more varied than turns in our study, just like in real navigation situations. While turns usually only differ between left and right, landmarks can be extremely varied (Caduff and Timpf 2008) and sometimes hard to define (Presson and Montello 1988). Relying on landmarks therefore requires more attention to the actual scenario than relying on turn information.

Explicit information about the direction source affected the linguistic expression of strategy choice through markers of mental as well as relational/passive processes. Without specific direction source information, words like *attention* and verbs like *figured, thought,* and *decide* were most frequent for participants using a turn-based strategy as opposed to all other participants (Fig. 8), while forms of *be* (representing relational or passive processes) were least frequent (Fig. 7). Thus, in the absence of explicit information about the direction source, the cognitive focus underlying a turn-based strategy was very much on the participants themselves and their thoughts, rather than on the scenario.

Switchers, in contrast, with their already enhanced focus on the scenario independent of condition (as discussed above), used a particularly high amount of relational/passive process terms in the absence of a cognitive focus on the direction source. Again, participants using a landmark-based strategy were in between these extremes, presumably for similar reasons related to the nature of landmarks in contrast to turns.

Interestingly, these patterns disappeared when the direction source was described as a specific human being or as a GPS system. In those cases, no differences between strategies could be found for relational/passive processes, but participants relying on landmark information used more markers indicating mental processes. They may have felt that they needed to additionally justify their thoughts and reasoning when the direction source was emphasized.

Altogether, these patterns suggest that even a slight shift in cognitive focus, either triggered by condition or by individual preferences, may result in judging situations differently and ultimately making different decisions in ambiguous navigation scenarios. This is in line with research showing that attention shifts can create biases not only with respect to how a scenario is perceived, but also how information is processed (Lepsien and Nobre 2006). In our study, the perceptual information as such was fairly limited, but how the situation was judged depended on the participants’ cognitive focus, guided by various factors such as previous information and individual preferences.

The logic behind most navigation decisions made in our study, as revealed by the content of retrospective reports, seemed clear enough; very few participants claimed that they guessed or made random decisions. It is a well-established fact that people draw on fast and frugal navigation heuristics (Todd and Gigerenzer 2000) that help them cope with diverse types of challenges, often influenced by specific features of the spatial scenario (Conlin 2009). Our study adds to these insights by showing that the specific situation proved to be relevant only to the extent that it was within the participant’s cognitive focus; others focused attention on aspects more closely related to their own experience and mental reasoning. This result contrasts to some extent with studies suggesting that generic questionnaires can probe stable individual preferences (Lawton 1996). Beyond our previous result showing that information about the direction source can trigger fundamental strategy shifts (Brunyé et al. 2015), we can now confirm that these strategy shifts are associated with fundamental shifts in cognitive focus that lead people to consider some factors as more decisive than others. Nevertheless, our data also suggest that at least some participants had individual preferences that remained stable.

The importance of attentional focus for decision making and other cognitive processes has long been recognized (e.g., Gonzalez et al. 2003; March and Shapira 1992). In communicative contexts, relevance for a person in a specific situation guides cognitive focus; sharing this sense of relevance allows for mutual understanding even in cases where messages are underspecified or ambiguous (Sperber and Wilson 1986). In spatial situations, the use of specific terms such as prepositions can reflect selective attention and perspectivization (Carstensen 2015). In our study, participants had to decide which aspects of the ambiguous message were relevant for their decision; different perceived communication sources as well as strategy preferences shifted their focus of attention in various ways. Since language is a powerful way of representing attentional focus (Marchetti et al. 2015; Talmy 2007), linguistic patterns in retrospective reports allowed us to trace attention shifts in ways related to the participants’ behavioral results, even when those shifts were not consciously revealed or readily identified in discourse.

## Conclusion

Nearly all navigation decisions rely on landmark and/or turn information. If landmark and turn information conflict in route directions, navigators are left uncertain and must decide which information to use. Our study shows that such decisions are associated with patterns of cognitive focus, triggered by situational features, such as information about the direction source and personal preferences to rely on either landmarks or turns.

In our experimental scenario, people could focus attention on the direction source, aspects of the situation, or on themselves (navigators) and their reasoning processes. The patterns of how people distributed attention could be assessed by analyzing relevant linguistic features in the participants’ retrospective reports. Results were systematically related to the participants’ navigation choices. People who switched strategies referred extensively to the scenario, whereas people who relied on turns referred much more to themselves, and people who relied on landmarks fell in between the two extremes. Altogether, our linguistic analysis of verbal representations highlights the decisive role of cognitive focus for spatial decisions made under uncertainty. GPS systems guide our attention toward turn directions, in spite of our natural ability to orient toward landmarks—leaving us to puzzle over the right decision in cases of uncertainty. It may be worthwhile taking a moment to consider landmark locations next time we’re unsure.
